# Comparative Study Among Ketamine, Fentanyl, and Ropivacaine, as Pre-incisional Analgesic Given by Surgical Site Infiltration, in Cases Posted for Elective Lower Segment Cesarean Section Under General Anesthesia

**DOI:** 10.7759/cureus.13609

**Published:** 2021-02-28

**Authors:** Prashant Mishra, Jaybrijesh Yadav, Shubham Rai, Rakesh Bahadur Singh

**Affiliations:** 1 Anaesthesiology, Rama Medical College & Hospital, Ghaziabad, IND; 2 Anaesthesiology, Uttar Pradesh University of Medical Sciences, Etawah, IND

**Keywords:** fentanyl, ketamine, ropivacaine, surgical site infiltration, apgar, vas, rss

## Abstract

Aim

The aim of this study was to evaluate the effect of surgical site infiltration prior to giving incision, with ketamine, fentanyl, and ropivacaine, on perioperative hemodynamic parameters, pain, and any adverse outcomes.

Methods

The study was carried out on 68 patients divided into four groups of 17 each. After intubation, the study drug was infiltrated subcutaneously at the surgical site, seven min before incision, by the surgeon who was unaware of the injectate. Hemodynamic variables were monitored and recorded during the intraoperative period and postoperative period at one, two, four, six, eight, 12, and 24 hours. The level of sedation was assessed using the Ramsay sedation score (RSS). The fetal outcome was assessed by the APGAR (Appearance, Pulse, Grimace, Activity, and Respiration) score at one, five, and 10 minutes.

Results

The mean visual analog scale (VAS) scores were significantly (p < 0.05) lower in group A as compared to other groups for most of the postoperative period. Hemodynamic stability was also significantly better with group A as compared to other groups during the perioperative period. The fetal outcome was comparable between the study groups with the APGAR scores similar between the groups at 10 min. However, significantly lower APGAR values were seen in group C as compared to the other study groups, at one and five minutes.

Conclusion

Ketamine infiltration resulted in a better hemodynamic profile, a significantly longer duration of the postoperative pain-free interval, and more sedation in the postoperative anesthesia care unit (PACU). It did not affect the fetal wellbeing, as discernable from the APGAR scores.

## Introduction

Cesarean section (CS) is the most common surgery carried out on women worldwide [1]. Regional anesthesia (RA) is the anesthesia of choice for CS. However, conditions sometimes necessitate the use of general anesthesia (GA) for providing anesthesia in patients posted for CS. Opioids form the backbone of analgesia in GA. However, their use is limited in CS for fear of placental transfer to the fetus and an increased chance of fetal depression. This may lead to a condition, wherein the patient may encounter significant pain in the intraoperative and postoperative periods. Another strategy for relief in incisional and postoperative pain involves the infiltration of the surgical wound with local anesthetic drugs. Preoperative wound infiltration as the method of preemptive analgesia was first addressed as early as 1978 [2]. The concept of preemptive or pre-incisional analgesia focuses on the prevention of central sensitization triggered by surgical incision; however, other factors have been advocated to exaggerate acute and long‑term postoperative pain as a result of central sensitization. These include noxious intraoperative stimuli as retraction, postoperative inflammatory processes, and ectopic neural activity [3]. Watanabe et al. studied retrospectively and showed that fentanyl, by continuous subcutaneous infusion, is a useful alternative for cancer pain [4]. Tan et al. found that pre-incisional treatment with subcutaneous infiltration of ketamine prolongs the time to the first analgesic requirement and decreases the total dosage of analgesics used and pain score after circumcision surgery [5]. Local wound infiltration with local anesthetics has been suggested to decrease perioperative opioid consumption and postoperative pain [6-7]. Divecha NP et al. concluded that ropivacaine infiltration was more effective for postoperative pain control with better results when done before incision [8]. It has significantly decreased pain intensity and diclofenac sodium consumption and has delayed the first rescue requirement. Although a number of studies have been conducted using ropivacaine, ketamine, and fentanyl as pre-emptive analgesics by surgical site infiltration in various surgeries.

## Materials and methods

This is a prospective, randomized (computer-generated), double-blind study. The study was undertaken after approval from the ethics committee, UPUMS, Saifai. Informed and written consent was taken and the study was carried out on 68 patients (sample size calculation was based on a population standard deviation of 1.1 with 80% power and 5% alpha error and a confidence level of 95%) divided into four groups of 17 each, age group 18-40 years, body mass index (BMI) 18-29 kg/m^2^, American Society of Anaesthesiologists physical classification I and II (ASA I and II) posted for elective lower segment cesarean section under general anesthesia. Group A (n=17) received infiltration at the surgical site with inj ketamine 1.5 mg/kg diluted up to 20 ml, Group B (n=17) received infiltration at the surgical site with inj ropivacaine 0.5% 20 ml, Group C (n=17) received infiltration at the surgical site with inj fentanyl 100 μg diluted up to 20 ml, Group D (n=17) received infiltration at the surgical site with inj normal saline 20 ml. In the operating theater (OT) standard monitors were attached and a baseline reading of heart rate (HR), systolic blood pressure (SBP), diastolic blood pressure (DBP), mean arterial pressure (MAP), and peripheral oxygen saturation (SPO2) was recorded. After three minutes of preoxygenation with 100% O2, patients were induced with inj. thiopentone sodium 5 mg/kg body weight and relaxed with inj. succinylcholine chloride 1.5 mg/kg body weight. Intubation was performed by an experienced anesthetist. Anesthesia was maintained with 0.6% isoflurane in 50% nitrous oxide with 50% oxygen mixture. Seven min before incision, the study drug was infiltrated subcutaneously at the surgical site by the surgeon who was not involved in the study. Hemodynamic variables were monitored and recorded during the intraoperative period. After the completion of surgery patients were shifted to the post-anesthesia care unit (PACU), where the postoperative pain was assessed using a visual analog score (VAS) = 0-10. Hemodynamic parameters were recorded at one, two, four, six, eight, 12, and 24 hours after completion of surgery. Inj diclofenac sodium 75 mg intravenous (IV) was administered as rescue analgesia when VAS score equal to or more than 5 were recorded. The level of sedation was assessed using the Ramsay Sedation Score (RSS). The fetal outcome was assessed by the APGAR (Appearance, Pulse, Grimace, Activity, and Respiration) score at 1, 5, and 10 minutes. All data were compiled systematically and analyzed using mean and standard deviation. Age, body mass index (BMI), duration of surgery, and hemodynamic variables were analyzed using analysis of variance (ANOVA) with post-hoc Bonferroni test. VAS scores, Ramsay Sedation Score, and APGAR scores were compared using the chi-square test. Microsoft Excel (Microsoft Corporation, Redmond, WA) and SPSS version 22 (IBM Corporation, Armonk, NY) software packages were used for data entry and analysis respectively. P-value < 0.05 is considered statistically significant.

## Results

Table 1 shows the demographic profile and duration of surgery among groups. There was no significant (p>0.05) difference in age, body mass index, and duration of surgery among the groups, showing the comparability of the groups in terms of age, BMI, and duration of surgery.

**Table 1:** Demographic profile and duration of surgery among groups BMI: body mass index

	Group A Mean±SD (n=17)	Group B Mean±SD (n=17)	Group C Mean±SD (n=17)	Group D Mean±SD (n=17)	p-value
Age (years)	26.65±3.32	27.94±4.72	26.53±2.65	24.76±3.23	0.210
BMI (kg/m^2^)	23.88±2.11	24.03±2.39	23.32±2.40	23.66±2.05	0.808
Duration of surgery (minutes)	52.12±7.15	49.88±5.66	52.24±4.59	52.47±6.35	0.565

Table 2 shows the intergroup comparison of intraoperative heart rate across time. The intergroup comparison shows that the HR at 0 min after infiltration of the study drug was higher in group D as compared to other groups and is significant (p < 0.05). During the intergroup comparison, the HR at the time of incision (7 min) was found to be significantly higher in group D as compared to groups B and C. During the intergroup comparison, the HR at eight min (1 min after incision) and 10 min was observed to be significantly lower in group C as compared to group D. The HR at 20 min was found to be significantly (p < 0.05) higher in groups C and D as compared to group B and was significantly lower in group C as compared to group A. The HR after extubation was observed to be significantly (p < 0.05) higher in group D as compared to groups A, B, and C.

**Table 2:** Shows the intergroup comparison of intraoperative heart rate across time periods HR: heart rate

Heart rate (beats per minute)		p-value					
		Group A vs B	Group A vs C	Group A vs D	Group B vs C	Group B vs D	Group C vs D
Baseline		1.000	1.000	0.113	1.000	0.212	0.232
After Intubation		1.000	1.000	0.064	1.000	0.086	0.879
After infiltration of study drug	0 Min	1.000	1.000	0.000	1.000	0.000	0.000
	7 min (time of incision)	0.289	0.824	1.000	1.000	0.011	0.048
	8 min (1 min after incision)	1.000	0.104	0.957	0.760	0.141	0.002
	10 Min	1.000	0.220	1.000	0.235	1.000	0.024
	20 Min	1.000	0.040	0.057	0.006	0.009	1.000
	30 Min	1.000	1.000	1.000	1.000	1.000	1.000
	40 Min	1.000	1.000	1.000	0.462	1.000	1.000
HR after extubation		0.857	1.000	0.000	0.165	0.046	0.000

Table 3 shows the comparison of mean arterial pressure between the groups during the intraoperative period. The MAP at 0 min after infiltration of the study drug was significantly (p < 0.05) higher in group D as compared to groups A, B, and D and significantly (p < 0.05) lower in group B as compared to group C. MAP at the time of incision (7 min) was found to be significantly higher in group A as compared to group B and D. MAP at 20 min was significantly (p < 0.05) lower in group B as compared to other groups. The MAP at 30 min being significantly (p < 0.05) lower in group D as compared to group C, at 40 min to be significantly (p < 0.05) lower in group D as compared to group C. The MAP after extubation was significantly (p < 0.05) lower in group A and B as compared to group D, as seen in intergroup comparison.

**Table 3:** Shows the intergroup comparison of the intraoperative mean arterial pressure across time periods

		p Value^1^					
		Group A vs B	Group A vs C	Group A vs D	Group B vs C	Group B vs D	Group C vs D
Baseline		1.000	1.000	0.191	1.000	0.388	1.000
After Intubation		1.000	1.000	0.110	0.920	0.063	1.000
After infiltration of study drug	0 Min	0.255	1.000	0.000	0.023	0.000	0.000
	7 min (time of incision)	0.018	1.000	0.021	0.192	1.000	0.212
	8 min (1 min after incision)	0.685	0.354	0.109	1.000	1.000	1.000
	10 Min	0.331	0.926	0.651	1.000	1.000	1.000
	20 Min	0.029	1.000	1.000	0.011	0.029	1.000
	30 Min	1.000	1.000	0.461	1.000	0.423	0.013
	40 Min	1.000	0.207	1.000	0.568	0.661	0.009
After Extubation		1.000	1.000	0.010	1.000	0.010	0.061

Table 4 shows the intergroup comparison of HR and mean arterial pressure across time periods among the groups during the postoperative period. HR at one hour was significantly (p < 0.05) higher in group D as compared to other groups, at two hours was significantly (p < 0.05) lower in group A as compared to groups C and D, at four hours was significantly (p < 0.05) higher in group B as compared to other groups, at six hours being significantly (p < 0.05) higher in group B as compared to groups C and D. HR at time of rescue analgesia was significantly (p < 0.05) higher in groups B and D as compared to groups A and C. Intergroup comparison shows that MAP at one hour was significantly (p < 0.05) higher in group D as compared to other groups, MAP at two hours was significantly (p < 0.05) lower in group A as compared to groups C and D and significantly (p < 0.05) lower in group B as compared to group C, at four hours significantly (p < 0.05) higher in group B as compared to other study groups, at six hours was significantly (p < 0.05) higher in group A as compared to group D. MAP at the time of rescue analgesia was noticed to be significantly (p < 0.05) lower in group A as compared to group D.

**Table 4:** Shows the intergroup comparison of postoperative heart rate and mean arterial blood pressure across time periods

	Time	p-value					
		Gr A Vs B	Gr A Vs C	Gr A Vs D	Gr B Vs C	Gr B Vs D	Gr C Vs D
	1 Hour	1.000	1.000	0.000	1.000	0.000	0.000
Postop. heart rate	2 Hour	0.187	0.025	0.016	1.000	1.000	1.000
	4 Hour	0.000	1.000	1.000	0.000	0.000	1.000
	6 Hour	1.000	0.658	0.308	0.024	0.008	1.000
	8 Hour	1.000	1.000	0.680	1.000	1.000	1.000
	12 Hour	1.000	1.000	1.000	1.000	1.000	1.000
	24 Hour	1.000	1.000	0.490	1.000	0.872	0.725
	At rescue analgesia	0.000	1.000	0.000	0.000	0.004	0.000
	1 Hour	1.000	0.131	0.000	0.007	0.000	0.002
Postop. mean BP	2 Hour	1.000	0.000	0.016	0.001	0.206	0.334
	4 Hour	0.000	1.000	1.000	0.001	0.000	0.161
	6 Hour	1.000	0.470	0.044	1.000	0.393	1.000
	8 Hour	1.000	1.000	1.000	1.000	1.000	1.000
	12 Hour	0.946	1.000	1.000	1.000	0.053	0.703
	24 Hour	0.240	1.000	1.000	0.462	0.075	1.000
	At rescue analgesia	0.207	0.567	0.002	1.000	0.628	0.233

Table 5 shows the intergroup comparison of VAS across the time period during the postoperative period. During the intergroup comparison, VAS at 0 hours was significantly (p < 0.05) lower in groups A and B as compared to groups C and D and significantly (p < 0.05) lower in group C as compared to group D, at one hour significantly lower in groups A and B as compared to other groups, at two hours significantly (p < 0.05) lower in groups A and B as compared to groups C and D and significantly lower in group A as compared to group B, at four hours was significantly lower in group A as compared to other groups and significantly (p < 0.05) higher in group B as compared to groups C and D, at six hours was significantly lower in groups B and C as compared to group D, at eight hours to be significantly higher in group D as compared to other groups, at 12 hours was found to be significantly lower in group A as compared to groups C and D, at 24 hours significantly lower in group A as compared to groups C and D.

**Table 5:** Shows intergroup comparison of VAS across time periods in the postoperative period VAS: visual analog score

	p-value					
	Group A vs B	Group A vs C	Group A vs D	Group B vs C	Group B vs D	Group C vs D
0 hour	0.060	0.000	0.000	0.040	0.000	0.000
1 hour	0.004	0.000	0.000	0.020	0.000	0.000
2 hour	0.003	0.000	0.000	0.001	0.005	0.569
4 hour	0.014	0.019	0.016	0.001	0.019	0.087
6 hour	0.229	0.122	0.059	0.030	0.045	0.000
8 hour	0.060	0.071	0.000	0.732	0.001	0.002
12 hour	0.083	0.038	0.000	0.724	0.008	0.004
24 hour	0.368	0.048	0.003	0.338	0.027	0.014
Time of rescue analgesia	0.480	0.193	0.193	0.310	0.310	*

Table 6 shows the intergroup comparison of RSS across time periods in the postoperative period. According to the intergroup comparison, RSS at 0 hours was significantly (p < 0.05) higher in group A as compared to other groups. At two hours, RSS was 2.00±0.00 in all groups. No statistics could be computed because RSS was a constant. RSS values were 2.00±0.00 in all groups at six, eight, 12, and 24 hours. RSS at the time of rescue analgesia was significantly (p < 0.05) higher in groups A and C as compared to groups B and D.

**Table 6:** Shows the intergroup comparison of RSS across time periods in the postoperative period RSS: Ramsay Sedation Scores

Time	P value					
	Group A vs B	Group A vs C	Group A vs D	Group B vs C	Group B vs D	Group C vs D
0 hour	0.015	0.037	0.015	0.310	*	0.310
1 hour	*	0.310	*	0.310	*	0.310
2 hour	*	*	*	*	*	*
4 hour	0.310	*	*	0.310	0.310	*
6 hour	*	*	*	*	*	*
8 hour	*	*	*	*	*	*
12 hour	*	*	*	*	*	*
24 hour	*	*	*	*	*	*
Time of rescue analgesia	0.015	1.000	0.015	0.015	*	0.015

Table 7 shows a comparison of APGAR scores between groups. The APGAR scores were found to be significantly lower (p < 0.05) in group C as compared to other study groups, at one and five min, according to the intergroup comparison. At 10 min, the mean APGAR scores did not differ significantly (p > 0.05) from a statistical point of view.

**Table 7:** Shows intergroup comparison of APGAR scores across time periods APGAR: Appearance, Pulse, Grimace, Activity, and Respiration

	p-value					
	Group A vs B	Group A vs C	Group A vs D	Group B vs C	Group B vs D	Group C vs D
1 Min	0.756	0.001	0.726	0.005	0.397	0.000
5 Min	0.217	0.002	0.403	0.028	0.659	0.007
10 Min	0.528	0.415	0.486	0.939	0.587	0.589

## Discussion

The present study aims to study the effect of pre-incisional surgical site infiltration of ketamine, ropivacaine, and fentanyl on incisional pain with the primary objective being the assessment of the postoperative pain in the patients using time until use of rescue analgesia and VAS. The secondary objectives of the study were to evaluate any difference between the groups in relation to hemodynamic stability, level of sedation, and fetal outcome. Abdallah NM et al., in their study, assessed the analgesic effectiveness of the pre-incisional infiltration of ketamine following elective abdominal hysterectomy as compared to levobupivacaine [9]. In their study, there was no significant difference between the two groups with regards to blood pressure and heart rate in the postoperative period. However, in our study, there were significant differences between the groups in the context of MAP and HR. Sarhan NA et al. in their study compared MAP and heart rate between the groups receiving ketamine infiltration and the control group [10]. They found MAP at 20 and 30 minutes after injection to be significantly lower in groups receiving ketamine as compared to the control group. However, in our study, no significant difference was found between group A and group D, except at the time of drug infiltration and after extubation. In our study, VAS was significantly lower in the ketamine and ropivacaine group as compared to the other groups in the whole post-operative group, with the ketamine group performing better than the ropivacaine group at one, two, four, eight, and 12 hours. This is in solidarity with the study by Abdallah NM et al., in which they found that VAS in the ketamine group was significantly lower than that in the levobupivacaine group [9]. Safavi M et al., in their study, recorded that VAS scores in ketamine infiltrated groups were significantly lower than in the normal saline infiltrated group [11]. This runs in conjugation with our study. Siddiqui AS et al. also observed similar findings in their study, wherein they found the pain scores to be significantly higher in the group receiving normal saline infiltration in comparison to the groups receiving ketamine infiltration [12]. In our study, RSS at 0 hours was significantly higher in the group receiving ketamine as compared to other groups, indicating greater sedation in the ketamine-infiltrated patients. At the time of rescue analgesia, sedation scores were higher in groups A and C as compared to other groups. For most of the postoperative period, all groups reported an RSS of 2, extending from the first hour itself. Findings on a similar note were reported by El Bahnasawy NS et al. [13]. They reported that the studied groups achieved a score of 1 (awake and oriented) within one hour in the PACU. In the study by Sarhan NA et al., sedation was assessed postoperatively by the Wilson sedation scale [10]. All patients in the three study groups achieved a score of 1 (awake and oriented) within one hour in PACU. In our study, APGAR scores were significantly lower in the fentanyl group as compared to the other three groups at one and five minutes. But, the scores became significantly indistinct at 10 min. This occurs in conjugation with the meta-analysis by Heesen M et al. [14], who found that ketamine administration has no effect on the neonatal outcome. However, our findings contradict the observations by Karbasy SH et al., who, in their study, evaluated the effect of low-dose fentanyl as a premedication before the induction of general anesthesia on the neonatal APGAR score in cesarean section delivery [15]. They concluded that the administration of 1 μg/Kg intravenous fentanyl before the induction of anesthesia for cesarean section delivery does not have any effect on APGAR scores of the neonate in the first and fifth minutes after birth.

## Conclusions

Ketamine infiltration resulted in a better hemodynamic profile, a significantly longer duration of postoperative pain-free interval, more sedation in the postoperative anesthesia care unit (PACU), and did not affect the fetal wellbeing, as discernable from the APGAR scores.
